# Cu-MOF-Derived Nano-Dendritic Self-Supported Electrodes for Efficient Electrochemical Nitrate-to-Ammonia Conversion

**DOI:** 10.3390/molecules31132307

**Published:** 2026-07-01

**Authors:** Linfeng Qi, Yu’an Gao, Xiangyan Zhong, Yunxiang Liang, Shijing Yuan, Shaojun Yuan

**Affiliations:** Low-Carbon Technology & Chemical Reaction Engineering Laboratory, College of Chemical Engineering, Sichuan University, Chengdu 610065, China; yywstd13540892130@163.com (L.Q.); xixibiuxixi@163.com (Y.G.); zxy3077787400@163.com (X.Z.); 19048017360@163.com (Y.L.); scuysj2024@163.com (S.Y.)

**Keywords:** Cu-MOF, nano-dendritic, self-supported electrodes, electrochemical nitrate reduction, ammonia

## Abstract

Electrochemical nitrate reduction reaction (eNO_3_RR) has emerged as a promising alternative to the energy-intensive and carbon-intensive Haber–Bosch process for green ammonia synthesis. However, the intrinsic complexity of the eight-electron transfer pathway and inevitable competing side reactions limit the activity and selectivity of eNO_3_RR. Maximizing the utilization of active sites and ensuring structural stability in electrocatalysts are essential for promoting surface proton-coupled electron transfer and improving Faradaic efficiency. Herein, we present a copper metal–organic framework (Cu-MOF)-derived electrocatalyst synthesized via in situ electrosynthesis on copper foam, using cetyltrimethylammonium bromide (CTAB) as a structure-directing agent, followed by electroreduction to produce a self-supported, nano-dendritic structure. This three-dimensional architecture exposes abundant active sites and facilitates electron transport, enabling efficient nitrate-to-ammonia conversion. The optimized CTAB-assisted electrode achieves an ammonia yield of 14.33 ± 0.61 mg h^−1^ cm^−2^ with a Faradaic efficiency of 90.95 ± 2.28% at −1.7 V versus Ag/AgCl. This study introduces a versatile design strategy for copper-based electrocatalysts that integrates structural stability with high activity, offering a sustainable approach for both ammonia production and nitrate remediation.

## 1. Introduction

Ammonia (NH_3_) is a fundamental compound in the global chemical industry, serving as a critical feedstock for fertilizers, fine chemicals, refrigeration, and pharmaceuticals. Beyond its industrial significance, ammonia has recently gained attention as a promising carbon-free hydrogen carrier owing to its high gravimetric hydrogen content (17.6 wt%), high energy density, and ease of storage and transport [1,2,3,4]. However, the conventional Haber–Bosch process, which operates at 300–500 °C and 200–300 atm, is highly energy intensive and environmentally detrimental, contributing approximately 1–2% of global CO_2_ emissions due to its dependence on fossil-fuel-derived hydrogen [4,5,6,7,8,9]. Consequently, the development of sustainable and decentralized ammonia synthesis pathways under ambient conditions remains an urgent scientific and environmental challenge.

The electrochemical nitrogen reduction reaction (eNO_3_RR) offers a sustainable pathway for ammonia synthesis; however, its efficiency is severely constrained by the strong N≡N triple bond (941 kJ mol^−1^), the low solubility of N_2_ in aqueous electrolytes, and the competing hydrogen evolution reaction (HER), which undermines both activity and selectivity [10,11,12]. In contrast, nitrate (NO_3_^−^), an oxidized nitrogen species abundantly found in wastewater from agricultural runoff and industrial effluents [13,14,15,16], provides a more reactive and soluble nitrogen source for ammonia production. The electrochemical conversion of nitrate to ammonia (NO_3_^−^ + 9H^+^ + 8e^−^ → NH_3_ + 3H_2_O) not only facilitates green NH_3_ synthesis [17,18] but also alleviates nitrate pollution, a major contributor to eutrophication and severe health risks such as methemoglobinemia (“blue baby syndrome”) and cancer [19]. Conventional physicochemical nitrate removal methods, including ion exchange, electrodialysis, and reverse osmosis, although effective, are costly and generate secondary waste [20,21,22]. Therefore, eNO_3_^−^RR driven by renewable electricity has emerged as an appealing dual-function strategy for sustainable ammonia production and environmental remediation [23,24].

Despite its promise, the eNO_3_^−^RR involves a complex eight-electron, nine-proton transfer process with multiple intermediates (NO_2_^−^, NO, N_2_O, and N_2_), resulting in competing pathways and low selectivity toward NH_3_ [25,26]. Although noble metals such as Pt, Rh, and Ru exhibit excellent catalytic activity and selectivity, their high cost and limited availability hinder large-scale application [27,28]. In contrast, copper-based catalysts have emerged as efficient, earth-abundant alternatives capable of suppressing HER and facilitating nitrate reduction under ambient conditions [29,30,31]. However, metallic Cu is susceptible to oxidation and structural degradation during catalysis [32], leading to performance instability and diminished NH_3_ yield. Metal–organic frameworks (MOFs) offer a versatile platform for constructing nanostructured electrocatalysts owing to their tunable porosity, well-defined metal–ligand coordination, and thermal transformability [33,34]. Recent studies have demonstrated that Cu–MOF-derived materials can act as highly active precursors for eNO_3_^−^RR and other electrocatalytic reactions, such as CO_2_ reduction and methanol oxidation [35,36,37]. However, most previous investigations have focused on activity enhancement without fully addressing issues of structural stability, electron transfer efficiency, and the precise identification of active sites, which remain critical challenges for the rational design of next-generation Cu–MOF-derived catalysts.

**Figure 1:**
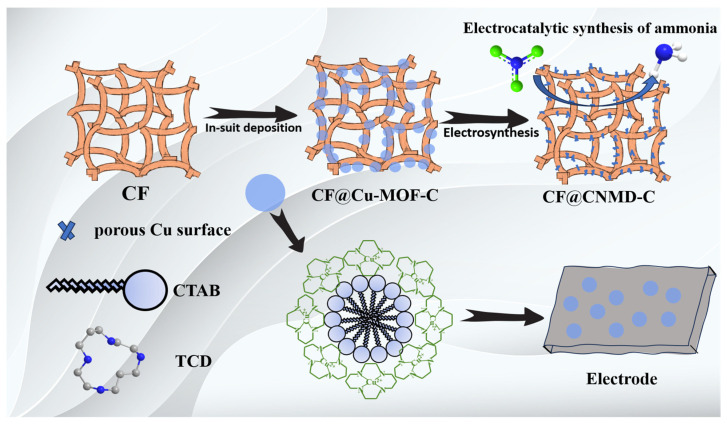
Schematic illustration of the in situ electrosynthesis of Cu-MOF precursors on copper foam with and without CTAB, forming CF@Cu-MOF-C and CF@Cu-MOF, respectively, followed by electrochemical reduction to yield Cu-MOF-derived nano-dendritic self-supported electrodes (CF@CMND-C and CF@CMND, respectively).

Herein, this study aims to fabricate a robust Cu–MOF-derived nano-dendritic (CF@CNMD) self-supported electrode. Inspired by the electrochemically assisted self-assembly (EASA) strategy proposed by Walcarius et al. [38,39], Cu-MOF thin films (CF@Cu-MOF-C) were in situ electrosynthesized on copper foam using CTAB as a structure-directing agent, as schematically illustrated in Figure 1. It is noteworthy that surfactant-based supramolecular templating, together with the synergistic interaction between ligands and surfactants, plays a crucial role in the cooperative mechanism leading to the formation of mesophase intermediates, followed by MOF growth and crystallization [40,41,42]. Specifically, electrostatic interactions between the negatively charged 1,4,8,11-tetrazacyclotetradecane (TCD) ligands and the positively charged quaternary ammonium headgroups of CTAB are expected to induce the formation of a mesophase structure on the electrode surface. Meanwhile, coordination between electron-donating TCD ligands and electron-accepting Cu^2+^ ions governs the growth of the Cu-MOF, thereby promoting the formation of a uniform thin-film Cu-MOF architecture on the copper foam substrate. Subsequent electrochemical reduction was performed to yield a stable Cu–MOF-derived framework with enhanced electrical conductivity, abundant exposed active sites, and improved electron transport properties. This CTAB-assisted in situ electrosynthesis strategy represents a novel approach for constructing hierarchically porous self-supported Cu-based electrodes with superior structural and electronic characteristics. The resulting architecture enables efficient and selective electrochemical nitrate-to-ammonia conversion under ambient conditions. Moreover, the CTAB-modified Cu-MOF-derived electrode exhibits markedly enhanced catalytic activity, selectivity, and stability compared with Cu foam and unmodified Cu-MOF electrodes, demonstrating a significant advancement in the rational design of Cu-based catalysts for sustainable ammonia synthesis. As a consequence, this study provides a new design paradigm for developing hierarchically structured, durable, and cost-effective copper-based catalysts for electrocatalytic nitrate reduction.

## 2. Results and Discussion

### 2.1. Morphological and Structural Analysis of the CF@CNMD-C Electrodes

As schematically illustrated in Figure 1, the CF@CNMD electrode was prepared via a two-step process: in situ electrodeposition of the Cu-MOF precursor followed by electrochemical reduction. During the anodic deposition stage, copper foam (CF) served as the anode in the electrolytic cell, where a positive potential induced the oxidation of copper, generating Cu^2+^ ions in the electrolyte. These Cu^2+^ ions coordinated with 1,4,8,11-tetrazacyclotetradecane (TCD) in the presence of CTAB to form Cu-MOF architectures (CF@Cu-MOF-C). The introduction of CTAB during synthesis modulated crystal growth at the electrode surface through its hydrophobic moieties, thereby controlling the three-dimensional morphology of the resulting Cu-MOF-C crystals. In the subsequent electrochemical reduction stage, the Cu-MOF-C precursor was reduced under constant current in a three-electrode system, producing stable, hierarchical, three-dimensional Cu-MOF-derived nano-dendritic (CF@CNMD-C) self-supported electrodes.

**Figure 2:**
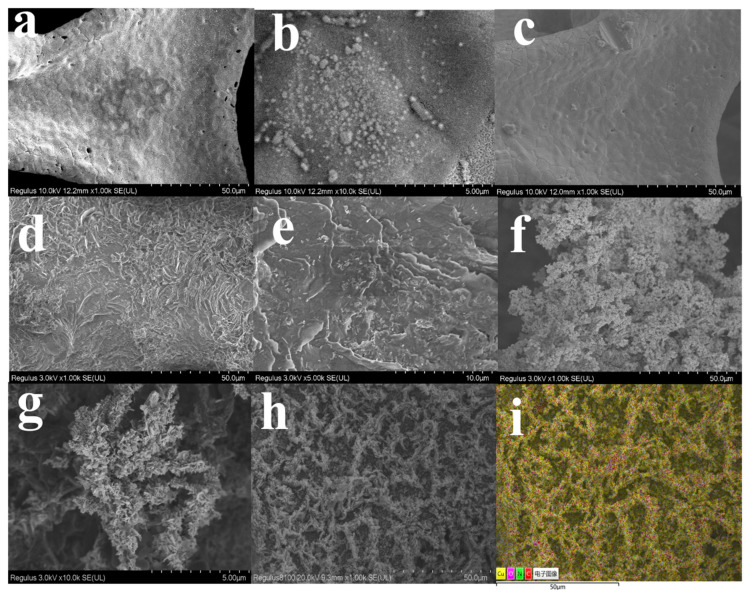
(**a**,**b**) SEM images of CF@Cu-MOF; (**c**) SEM image of CF@CNMD; (**d**,**e**) SEM images of CF@Cu-MOF-C, showing the three-dimensional interconnected and hierarchical nanostructure; (**f**–**h**) SEM images of CF@CNMD-C, illustrating the dendritic morphology and highly porous surface after electroreduction; (**i**) EDS overlay mapping corresponding to Figure 2h. The Chinese meaning in (**i**) is electronic image.

As shown in Figure 2a,b and Figure S1, the CF@Cu-MOF synthesized without CTAB exhibits a relatively smooth surface with only a few sparsely distributed crystals. After electroreduction, CF@Cu-MOF is transformed into CF@CMND, which retains a uniform surface morphology without the formation of pronounced rough or porous structures (Figure 2c and Figure S1). In contrast, the introduction of CTAB significantly alters the morphology of the Cu-MOF precursor. As shown in Figure 2d and Figure S2, nanosheet- and flake-like structures are densely deposited on the copper foam skeleton of CF@Cu-MOF-C, forming a continuous yet rough and uneven coating layer. Higher-magnification SEM images further reveal abundant nanostructures distributed across the surface, resulting in a highly rough and porous morphology (Figure 2e and Figure S2c). Such a hierarchical architecture provides a favorable framework for the subsequent growth of three-dimensional dendritic copper structures. After electroreduction, CF@CMND-C retains the interconnected porous framework of the copper foam substrate (Figure S2d–f). Well-defined dendrite-like nanostructures are formed on the copper skeleton (Figure 2f and Figure S2), producing a highly rough and porous surface with a substantially larger surface area than that of CF@CMND. This hierarchical micro/nanostructure is expected to increase the electrochemically active surface area, expose more accessible catalytic sites, and facilitate electrolyte penetration and nitrate mass transport, thereby accelerating NO_3_^−^ reduction kinetics. The corresponding EDS spectrum (Figure S3) and elemental mapping images (Figure S4) confirm the presence of Cu, O, C, and N in CF@CMND-C. Notably, Cu is uniformly distributed throughout the copper foam framework, while C, N, and O are homogeneously dispersed across the selected region, indicating the successful and uniform incorporation of Cu-MOF-derived components onto the electrode surface. The uniform elemental distribution, combined with the interconnected dendritic morphology, is expected to enhance interfacial contact, facilitate charge transfer, and ultimately improve the electrocatalytic performance toward nitrate reduction.

**Figure 3:**
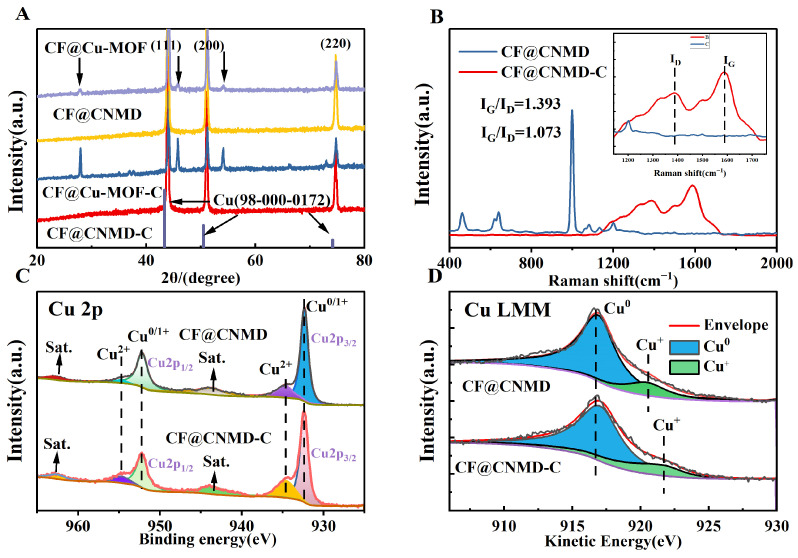
(**A**) XRD patterns of CF@Cu-MOF, CF@Cu-MOF-C, CF@CNMD, and CF@CNMD-C, illustrating the structural transformation from Cu-MOF to nano-dendritic metallic Cu after electroreduction. (**B**) Raman spectra of CF@CNMD and CF@CNMD-C, showing the D and G bands and the increased G/D ratio upon CTAB incorporation. (**C**,**D**) XPS spectra of CF@CNMD and CF@CNMD-C, revealing the Cu 2p core-level peaks and the surface valence states of Cu species.

The crystalline phases of Cu-MOF and its derivatives were characterized by XRD, as shown in Figure 3A. Both CF@Cu-MOF and CF@Cu-MOF-C exhibit a characteristic diffraction peak at 2θ of 27.9°, corresponding to the (004) crystal plane of Cu-MOF [43,44], without detectable impurity peaks, thereby confirming the successful in situ growth of Cu-MOF on the copper foam substrate. Notably, after the introduction of CTAB, the diffraction peaks of CF@Cu-MOF-C become more intense and well-defined, indicating enhanced crystallinity and increased Cu-MOF growth. This enhancement is attributed to the soft-template effect of CTAB, which regulates the nucleation and growth behavior of Cu-MOF during the in situ electrosynthesis process, thereby promoting the formation of a more uniform and ordered film-like structure on the copper substrate [45]. Combined with the SEM observations, these results further demonstrate the critical role of CTAB in regulating the morphological evolution of the material. After electrochemical reduction, the characteristic diffraction peaks of the pristine CF@Cu-MOF and CF@Cu-MOF-C nearly disappear. The XRD patterns of the CF@CNMD and CF@CNMD-C samples are dominated by three diffraction peaks at 43.3°, 50.4°, and 74.1°, corresponding to the (111), (200), and (220) crystal planes of metallic Cu (PDF #98-000-0172), respectively. These results indicate the successful electrochemical reduction of Cu-MOF to nano-dendritic metallic copper.

To further evaluate the structural ordering of the carbonaceous components, Raman spectroscopy was performed. As shown in Figure 3B, the Raman spectra of the samples exhibit a D band associated with disordered or defective carbon and a G band corresponding to the in-plane vibration of sp^2^-hybridized carbon atoms. After the introduction of CTAB, the G/D band area ratio increased from 1.073 for CF@CNMD to 1.393 for CF@CNMD-C, indicating enhanced graphitic ordering and a lower degree of structural disorder in the CTAB-modified sample. Previous studies have demonstrated that a lower I_D_/I_G_ ratio corresponds to a higher degree of graphitization and that CTAB can serve as an effective surfactant or structure-directing agent to promote the dispersion and exfoliation of graphene-based nanosheets [46,47,48]. In combination with the SEM and EDS results, these findings suggest that CTAB facilitates the formation and uniform distribution of graphene-like nanosheets, thereby enhancing electron transfer.

XPS characterization was performed to determine the surface compositions of CF@CNMD and CF@CNMD-C electrodes, and the results are shown in Figures S5–S7 and Figure 3C,D. The survey XPS spectra exhibit characteristic photoelectron signals corresponding to C 1s (285 eV), O 1s (532 eV), and Cu 2p (932 eV), confirming the successful formation of Cu-MOF-derived nano-dendritic layers on both CF@CNMD and CF@CNMD-C electrodes (Figure S5). The deconvoluted C 1s spectra can be assigned to C–C (284.6 eV) and O–C=O (288.2 eV) species, consistent with the TCD ligand structure in the Cu-MOF (Figure S6). Notably, the relative intensity of the O–C=O component in CF@CNMD-C is slightly higher than that in CF@CNMD, indicating a greater deposition of Cu-MOF-derived nano-dendritic layers in the presence of CTAB. This observation is further supported by the emergence of an additional -COOH peak at 535.8 eV in the deconvoluted O 1s spectrum on the CF@CNMD-C (Figure S7). As shown in Figure 3C, the deconvoluted Cu 2p core-level spectra of both samples exhibit characteristic spin–orbit doublets and satellite peaks, indicating the coexistence of Cu^2+^, Cu^+^, and Cu^0^ species. The peak centered with binding energy at 934.5 eV is assigned to Cu^2+^ species, while the peak at approximately 932.3 eV corresponds to Cu^+^/Cu^0^ species. However, Cu^+^ and Cu^0^ cannot be clearly distinguished solely from the Cu 2p spectra. Therefore, Cu LMM Auger spectra were further collected to clarify the valence-state distribution of the surface Cu species (Figure 3D). In comparison with CF@CNMD, the Cu^+^ species in CF@CNMD-C exhibit a positive shift in kinetic energy, indicating increased electron density around the Cu^+^ sites and a modified local electronic structure. This shift is attributed to the structural regulation induced by CTAB, which may alter the coordination environment of Cu species and strengthen the electronic interaction between Cu sites and the surrounding conductive matrix. Wang et al. reported that in situ generation of Cu_2_O active sites on island-like Cu significantly promotes the conversion of *HNOH to *HNHOH while simultaneously suppressing the competing hydrogen evolution reaction, highlighting the crucial role of Cu^+^/Cu_2_O-derived active sites in enhancing NH_3_ selectivity [49]. The electron-rich Cu^+^ sites are expected to facilitate nitrate adsorption and activation, as well as enhance interfacial electron transfer, thereby contributing to the improved electrocatalytic nitrate reduction performance of the CF@CNMD-C electrodes.

### 2.2. Electrochemical Properties of the CF@CNMD-C Electrode

**Figure 4:**
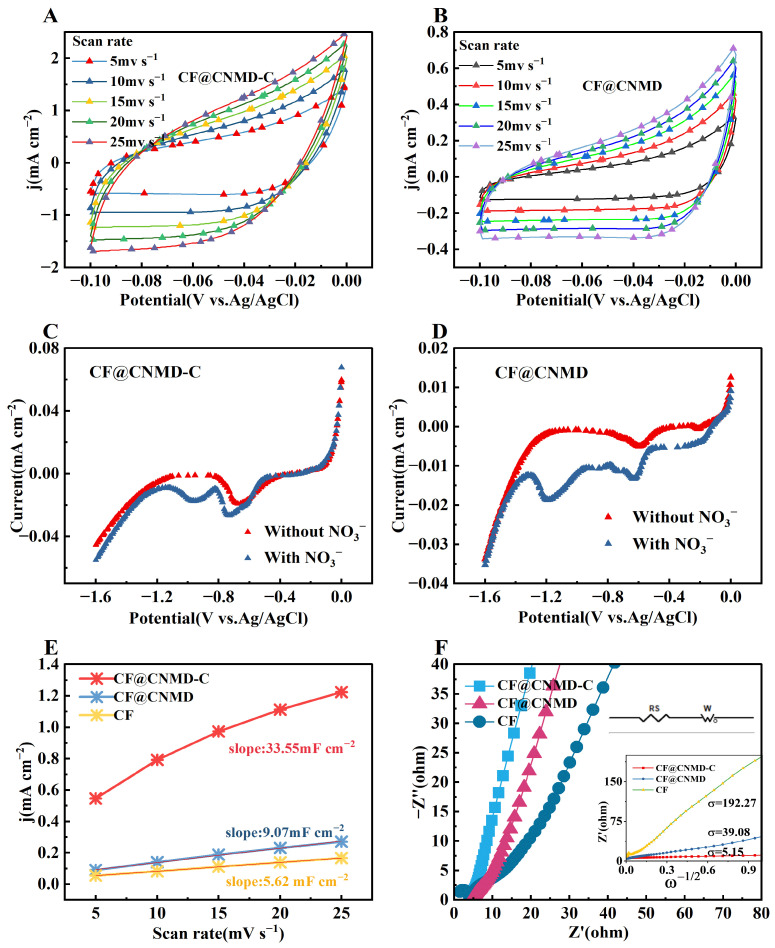
(**A**,**B**) Cyclic voltammetry (CV) curves of CF@CNMD-C and CF@CNMD at various scan rates for evaluating electrochemically active surface area. (**C**,**D**) Linear sweep voltammetry (LSV) curves of CF@CNMD-C and CF@CNMD in the presence and absence of nitrate, demonstrating electrocatalytic activity toward NO_3_RR. (**E**) Double-layer capacitance (C_dl_) values of CF@CNMD-C and CF@CNMD, indicating differences in electrochemically active surface area. (**F**) Nyquist plots and linear fitting plots of Z′ versus ω^−1/2^ in the low-frequency region from EIS of CF@CNMD-C and CF@CNMD, showing charge-transfer and ion diffusion behavior.

Electrochemical measurements, including LSV, CV, and EIS, were conducted to investigate the electrochemical properties of the CF, CF@CNMD, and CF@CNMD-C electrodes. The electrochemically active surface area (ECSA) was evaluated from CV measurements performed at scan rates of 5, 10, 15, 20, and 25 mV s^−1^ (Figure 4A,B and Figure S8). The double-layer capacitance (C_dl_) was determined by plotting the difference in capacitive current density against the scan rate. The ECSA was estimated according to the relationship

*ECSA = C_dl_* ∗ *A_geo_/C_s_*

where *C*_s_ is the specific capacitance of a smooth electrode surface, and *A*_geo_ is the geometric area of the electrode exposed to the electrolyte. In this work, *C*s = 40 μF cm^−2^ and *A*_geo_ = 1 cm^2^ were used. The value of *C*_dl_ is a commonly adopted parameter for estimating ECSA from double-layer capacitance in aqueous electrocatalytic systems and has been widely applied in previous studies, including the benchmark work by McCrory et al. [50]. Therefore, the calculated ECSA values of CF@CNMD-C, CF@CNMD, and CF are 838.75, 226.75, and 140.50 cm^2^, respectively. The ECSA of CF@CNMD-C is approximately 3.70 times higher than that of CF@CNMD and 5.97 times higher than that of bare CF. The CF@CNMD-C electrode exhibits a significantly larger ECSA value than those of the CF and CF@CNMD electrodes (Figure 4E), indicating a substantially increased electrochemically active surface area resulting from CTAB-assisted synthesis. This remarkable enhancement in ECSA is mainly attributed to the dendritic architecture and the higher degree of graphitization of CF@CNMD-C, consistent with the SEM and Raman analyses. Specifically, the dendritic morphology provides an open and highly accessible three-dimensional structure that exposes abundant electrochemically active sites and facilitates electrolyte penetration. Meanwhile, the improved graphitization degree enhances electron transport during the electrochemical process. Consequently, the increased ECSA of CF@CNMD-C is expected to promote nitrate adsorption, accelerate interfacial charge transfer, and ultimately enhance the nitrate reduction reaction.

LSV measurements were conducted at a scan rate of 5 mV s^−1^ to evaluate the electrocatalytic performance of the CF@CNMD and CF@CNMD-C electrodes toward nitrate reduction. As shown in Figure 4C,D, both electrodes exhibit relatively low cathodic current densities in 0.5 M Na_2_SO_4_ without NO_3_^−^ over the potential range from −0.6 to −1.6 V versus Ag/AgCl. Upon the addition of NO_3_^−^, the cathodic current densities increase markedly, indicating that the enhanced current response originates from electrochemical nitrate reduction. These results confirm that both electrodes possess electrocatalytic activity toward the nitrate reduction reaction.

To further investigate the charge-transfer behavior and ion diffusion kinetics during the catalytic process, EIS measurements were performed in 0.5 M Na_2_SO_4_, and the corresponding Nyquist plots are presented in Figure 4F. Notably, CF@CNMD-C exhibits a steeper slope in the low-frequency region compared with CF@CNMD and CF, indicating reduced ion diffusion resistance. This observation is further supported by the smaller slope obtained from the linear fitting of Z′ versus ω^−1/2^ for CF@CNMD-C, which corresponds to a lower Warburg coefficient [51], thereby confirming its superior ion diffusion capability (inset of Figure 4F). Furthermore, the CF@CNMD-C electrode displays a smaller charge-transfer resistance than the other electrodes, suggesting accelerated interfacial electron-transfer kinetics. These characteristics facilitate charge transport and mass transfer during electrolysis, thereby contributing to the enhanced electrocatalytic performance toward the nitrate reduction reaction.

As shown in the chronopotentiometry curves (Figure S9), all three electrodes exhibit an initial rapid potential change followed by stabilization in 0.5 M Na_2_SO_4_ containing 0.01 M KNO_3_. CF and CF@CNMD rapidly reach steady states at around −1.20 V vs. Ag/AgCl, indicating relatively stable but limited interfacial reconstruction during operation. In contrast, CF@CNMD-C displays a more pronounced initial potential evolution before stabilizing at a slightly more negative value (−1.30 V vs. Ag/AgCl), suggesting an activation process associated with surface reconstruction and formation of catalytically active sites. The stable long-term plateau further indicates robust interfacial integrity and efficient charge transport within the dendritic architecture. Overall, CF@CNMD-C exhibits enhanced interfacial activation behavior and electrochemical stability, consistent with its superior nitrate reduction performance.

### 2.3. Evaluation of eNO_3_RR Performance of the CF@CNMD-C Electrode

To evaluate the eNO_3_RR performance of the CF@CNMD and CF@CNMD-C electrodes, chronoamperometric electrolysis was performed in an electrolyte containing 0.01 M KNO_3_ and 0.5 M Na_2_SO_4_ for 1 h at various applied potentials. Following electrolysis, the catholyte was collected for the quantitative determination of NH_3_ and NO_2_^−^ using the standard curves shown in Figure S10. The NH_3_ yield and NO_2_^−^ concentrations were analyzed to assess the nitrate reduction performance of the electrodes and to identify the optimal operating potential.

**Figure 5:**
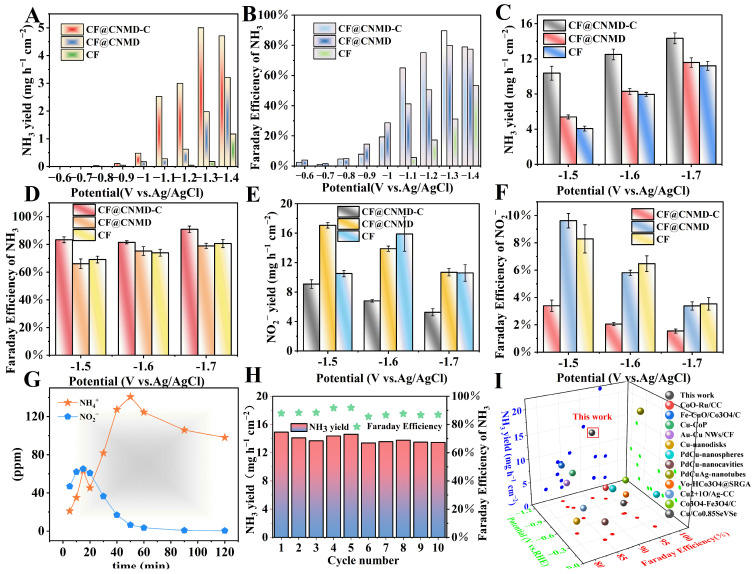
(**A**,**C**) Electrochemical nitrate reduction performance of CF, CF@CNMD, and CF@CNMD-C electrodes in 0.01 M KNO_3_ and 0.5 M Na_2_SO_4_ electrolyte. (**B**,**D**) NH_3_ yield and Faradaic efficiency of NH_3_ during chronoamperometric electrolysis at different applied potentials and durations, highlighting the enhanced ammonia production of CF@CNMD-C. (**E**,**F**) NO_2_^−^ yield and Faradaic efficiency, illustrating intermediate formation and selectivity trends for the three electrodes. (**G**) Time-dependent evolution of NH_3_ and NO_2_^−^ concentrations over CF@CNMD-C at −1.7 V vs. Ag/AgCl, demonstrating the kinetics of nitrate-to-ammonia conversion. (**H**) Cyclic stability of CF@CNMD-C at −1.7 V vs. Ag/AgCl over ten repeated cycles, showing excellent durability and reproducibility. (**I**) Comparative performance of CF@CNMD-C with previously reported electrocatalysts under the same electrolyte conditions, highlighting its superior NH_3_ yield and Faradaic efficiency.

As shown in Figure 5A,B, the CF, CF@CNMD, and CF@CNMD-C electrodes exhibit relatively low NH_3_ yield and Faradaic efficiencies (FE) within the potential range of −0.6 to −1.0 V versus Ag/AgCl. Nevertheless, the CF@CNMD-C electrode consistently achieves higher NH_3_ production rates and FE than the CF and CF@CNMD electrodes, indicating its superior intrinsic activity toward the NO_3_RR. As the applied potential becomes more negative, from −1.1 to −1.4 V versus Ag/AgCl, both the NH_3_ yield and FE increase markedly for all three electrodes. This behavior can be attributed to the increased availability of surface-adsorbed hydrogen species (*H) and accelerated interfacial electron transfer, which enhance the hydrogenation of nitrate-derived intermediates and facilitate the subsequent conversion of accumulated NO_2_^−^ intermediates into NH_3_ [52,53]. Notably, the CF@CNMD-C electrode attains a maximum NH_3_ yield rate of 5.004 mg h^−1^ cm^−2^ and a high FE value of 89.6% at −1.3 V versus Ag/AgCl, significantly outperforming the CF and CF@CNMD electrodes. These results demonstrate that CTAB-induced structural modulation effectively enhances both the eNO_3_RR activity and NH_3_ selectivity of the CF@CNMD-C electrode.

As shown in Figures S11 and S12, the NO_2_^−^ yield and FE were analyzed to elucidate the evolution of reaction intermediates during the eNO_3_RR. For CF@CNMD-C, the NO_2_^−^ yield increases as the applied potential shifts from −0.6 to −1.0 V versus Ag/AgCl, followed by a decline at more negative potentials. This trend suggests that NO_2_^−^ initially accumulates as a key reaction intermediate and is subsequently consumed through further reduction to NH_3_ under a stronger cathodic driving force [54]. The NO_2_^−^ yield reaches its maximum at −1.0 V versus Ag/AgCl, while the corresponding Faradaic efficiency peaks at −0.7 V and gradually decreases with further increases in cathodic potential. In comparison with CF and CF@CNMD electrodes, the earlier decline in both NO_2_^−^ yield and FE observed for the CF@CNMD-C electrode indicates more efficient conversion of NO_2_^−^ intermediates into NH_3_, which is consistent with its superior NH_3_ production performance.

Because this study focuses on the reduction of low-concentration nitrate, an electrolyte containing 0.01 M KNO_3_ was employed as the nitrate source for eNO_3_RR evaluation of the CF, CF@CNMD, and CF@CNMD-C electrodes. Within the potential range of −0.6 to −1.4 V versus Ag/AgCl, nitrate consumption becomes increasingly significant at potentials more negative than −1.1 V, which substantially influences the measured NH_3_ yield. Time-dependent experiments further reveal that the NH_3_ concentration begins to decrease after approximately 40–50 min of electrolysis, suggesting nitrate depletion and the occurrence of side reactions during prolonged operation. In addition, at more negative cathodic potentials, the hydrogen evolution reaction (HER) is intensified and competes with eNO_3_RR for electrons and adsorbed hydrogen species, thereby reducing the FE for NH_3_ production [55]. Therefore, the chronoamperometric electrolysis time was shortened from 1 h to 0.5 h for potentials ranging from −1.5 to −1.7 V versus Ag/AgCl to minimize the effects of nitrate depletion and HER interference, enabling a more accurate evaluation of NH_3_ production performance.

As depicted in Figure 5C–F, the ammonia production rates of all three electrodes increase with increasing cathodic potential. Notably, the CF@CNMD-C electrode achieves a high NH_3_ yield of 14.33 ± 0.61 mg h^−1^·cm^−2^ at −1.7 V versus Ag/AgCl, while maintaining an excellent Faradaic efficiency of 90.95 ± 2.28%. Although the CF@CNMD electrode exhibits higher NH_3_ production rates than the bare CF electrode, their NH_3_ FE values remain comparable, ranging from approximately 60% to 80%. In contrast, CF@CNMD-C consistently delivers substantially higher NH_3_ yield rates and FE values than CF@CNMD, demonstrating the beneficial effect of CTAB-induced structural modulation on eNO_3_RR performance. For the by-product NO_2_^−^, both the NO_2_^−^ yield and FE decrease with increasing cathodic potential for the CF@CNMD-C and CF@CNMD electrodes. Moreover, CF@CNMD-C exhibits lower NO_2_^−^ yields and FE than CF and CF@CNMD over the investigated potential range, indicating suppressed accumulation of the NO_2_^−^ intermediate and enhanced selectivity toward NH_3_ production. These results confirm the superior eNO_3_RR activity and selectivity of CF@CNMD-C, which are consistent with the structural characterization and electrochemical analyses discussed above.

Kinetic measurements and cyclic stability tests were further conducted on CF@CNMD-C electrodes to investigate time-dependent product evolution during chronoamperometric electrolysis and to evaluate long-term catalytic stability toward eNO_3_RR (Figure 5G,H). The kinetic analysis reveals the temporal evolution of NH_3_ and NO_2_^−^ concentrations. As shown in Figure 5G, NH_3_ concentration steadily increases during the initial stage, reaching a maximum after approximately 50 min, indicating efficient nitrate-to-ammonia conversion by CF@CNMD-C. Upon extended electrolysis, the NH_3_ concentration gradually decreases. This phenomenon is likely associated with the intensified hydrogen evolution reaction (HER). In our experiments, abundant gas bubbles were also observed on the electrode surface at the later stage of electrolysis, further indicating the enhanced HER. The formation and detachment of H_2_ bubbles may disrupt the local reaction environment, aggravate mass-transfer limitations, and induce pH fluctuations near the electrode surface. Such local pH changes can shift the NH_4_^+^/NH_3_ equilibrium toward volatile NH_3_, thereby promoting partial ammonia loss during prolonged electrolysis [56,57]. As shown in Figure 5H, cyclic stability tests demonstrate the excellent durability of the CF@CNMD-C electrode. After ten consecutive cycles, the NH_3_ yield rate remains within the range of 13.39–14.92 mg h^−1^ cm^−2^, with an average value of 13.94 mg h^−1^ cm^−2^, while the Faradaic efficiency varied from 85.53% to 91.81%, averaging 88.13%. Notably, the NH_3_ yield rate retains 90.28% of its initial value after ten cycles, confirming the good cycling stability and long-term electrocatalytic durability of the electrode for nitrate-to-ammonia conversion.

To further evaluate the structural stability of the electrode during long-term operation, Cu leaching into the electrolyte after the cycling tests was quantified by ICP–OES. As summarized in Table S2, the dissolved Cu concentration ranges from 0.2164 to 1.8540 mg L^−1^, corresponding to only 10.82–92.70 μg of Cu in 50 mL of electrolyte. The relatively low Cu dissolution indicates that severe electrode corrosion or Cu leaching does not occur during electrolysis, demonstrating the good structural stability of the Cu-based electrode. Furthermore, the morphology and phase composition of the electrode after ten consecutive cycles were examined by SEM and XRD (Figures S13 and S14). The SEM images reveal only minor morphological changes, with the dendritic microstructure and rough surface texture largely preserved after prolonged operation (Figure S13). XRD analysis shows that metallic Cu remains the dominant crystalline phase (Figure S14), although a small amount of Cu_2_O is detected, likely resulting from partial surface oxidation during repeated electrolysis cycles. These results collectively confirm the excellent structural robustness and durability of the CF@CNMD-C electrode under NO_3_^−^ reduction conditions.

To further highlight the superior NO_3_RR performance of the CF@CNMD-C electrode, its electrocatalytic activity was systematically compared with previously reported electrocatalysts under 0.01 M NO_3_^−^ similar experimental conditions, and the comparison plot is displayed in Figure 5I. Detailed information on the applied potential, Faradaic efficiency, NH_3_ yield rate, and energy consumption is summarized in Table S1. Notably, the CF@CNMD-C electrode exhibits superior or comparable performance in terms of both NH_3_ yield rate and Faradaic efficiency relative to previously reported electrocatalysts. These favorable metrics are achieved at a relatively moderate applied potential, highlighting the excellent overall activity and selectivity of the CTAB-assisted CF@CNMD-C electrode for nitrate electroreduction. Furthermore, previous studies by Kamyabi et al. [58], Ding et al. [59], and others [60] have demonstrated that CTAB-assisted or surfactant-directed architectures can enhance electrocatalytic performance by tailoring catalyst morphology, increasing the density of accessible active sites, and facilitating mass and charge transport. These findings are consistent with the present results and further support the critical role of CTAB-templated morphology in promoting catalytic activity. The energy consumption of CF@CNMD-C was estimated to be approximately 15.58 kWh kg_NH3_^−1^ and compared with representative eNO_3_RR catalysts reported in the literature. Although CF@CNMD-C does not exhibit the lowest energy consumption among the catalysts compared, it delivers a high NH_3_ production rate while maintaining excellent Faradaic efficiency, demonstrating its favorable overall ammonia production performance. Moreover, the catalyst is composed entirely of non-noble-metal components, offering additional advantages in terms of cost-effectiveness and practical scalability for large-scale nitrate-to-ammonia conversion.

## 3. Experimental Methods

### 3.1. Materials

Copper foam (Cu, >99% purity, 1 mm thickness) was obtained from Kesheng Foam Metal Co., Ltd. (Suzhou, China). Chemical reagents, including 1,4,8,11-tetrazacyclotetradecane (TCD, 98%), sodium nitroprusside (C_5_FeN_6_Na_2_O·2H_2_O, 99%), sodium sulfate (Na_2_SO_4_, 99.5%), hexadecyltrimethylammonium bromide (CTAB, C_19_H_42_BrN, >96%), sodium citrate dihydrate (C_6_H_5_Na_3_O_7_·2H_2_O, >99%), and salicylic acid (C_7_H_6_O_3_, 99.7%), were purchased from Shanghai Titan Technology Co., Ltd. (Shanghai, China). Additional reagents, including ammonium chloride (NH_4_Cl, >99.5%), sodium nitrite (NaNO_2_, >95%), potassium nitrate (KNO_3_, >99%), sodium hydroxide (NaOH, >98%), sulfanilamide (C_6_H_8_N_2_O_2_S, >95%), sodium hypochlorite solution (NaClO·5H_2_O, 30%), sulfamic acid (H_3_NO_3_S, >99.5%), hydrochloric acid (HCl, >37%), phosphoric acid (H_3_PO_4_, >85%), acetone (C_3_H_6_O, 99.5%), anhydrous ethanol (C_2_H_5_OH, >99.5%), and naphthalene ethylenediamine dihydrochloride (C_12_H_14_N_2_·2HCl) were purchased from Kelong Chemical Co., Ltd. (Chengdu, China). All chemicals were used as received without further purification. Deionized water (resistivity > 18.25 MΩ·cm) from an ultrapure water purification system was used in all experiments.

### 3.2. Preparation of CF@Cu-MOF on Copper Foam

Copper foam sheets (1 cm × 1 cm × 0.03 cm) were sequentially cleaned by ultrasonication in acetone, ethanol, and deionized water, each for 10 min, to remove surface contaminants. The cleaned foams were then immersed in 1 M HCl under ultrasonic agitation for 20 min to remove surface oxides, followed by a final rinse with deionized water for 10 min. The in situ electrosynthesis of CF@Cu-MOF precursors on copper foam was performed following a procedure adapted from previously reported MOF electrodeposition methods. Two pretreated copper foam sheets (10 mm × 10 mm) were used as the working and counter electrodes, separated by 1 cm. Electrochemical synthesis was conducted using a constant-current/constant-potential system (CS310, Wuhan Corrtest Instruments Co., Ltd., Wuhan, China). The electrolyte comprised 50 mL of a 1:1 (*v*/*v*) ethanol–water mixture containing 10 mg of TCD and 2 g of CTAB. A potential difference of 10 V was applied between the electrodes, and the reaction was maintained at 70 °C for 5 min. The resulting Cu-MOF-deposited copper foam was designated as CF@Cu-MOF-C. For comparison, Cu-MOF was also electro-synthesized in the absence of CTAB, yielding the CF@Cu-MOF sample.

### 3.3. Fabrication of CF@CNMD Self-Supported Electrode

The Cu–MOF-derived nano-dendritic self-supported electrodes with stable carbon-based structures and enhanced electrocatalytic properties were prepared via a well-established electroreduction method. In a typical procedure, CF@Cu-MOF (prepared without CTAB) or CF@Cu-MOF-C (prepared with CTAB) precursor electrodes were used as the cathode in an H-type electrolytic cell employing a three-electrode system, with a platinum sheet as the counter electrode and an Ag/AgCl electrode as the reference. A 0.5 M Na_2_SO_4_ aqueous solution served as the electrolyte to improve ionic conductivity. Electroreduction was performed under a constant cathodic current of 0.01 A for 5000 s. The resulting sample derived from CF@Cu-MOF-C was designated CF@CNMD-C, while the product from CF@Cu-MOF was denoted CF@CNMD.

### 3.4. Characterization

The crystal structure of the as-prepared samples was analyzed by X-ray diffraction (XRD) using a MiniFlex600 diffractometer (Rigaku, Tokyo, Japan) with Cu-Kα radiation (λ = 0.154056 nm) at 25 °C in a 2θ range of 10–80°. Morphology and elemental distribution were examined using scanning electron microscopy (SEM) and energy-dispersive spectroscopy (EDS) mapping on an FEI Quanta 250 (Regulus 8230U, Hitachi, Japan). Surface composition was determined by X-ray photoelectron spectroscopy (XPS, Kratos Analytical Ltd., Manchester, UK) equipped with a monochromatic Al Kα source (1486.6 eV). Surface defects and degree of graphitization were evaluated using a DXR Raman microscope (Thermo Fisher Scientific, Waltham, MA, USA) with an argon ion laser (λ = 455 nm). The concentrations of leached Cu^2+^ ions in the electrolyte were determined using an Optima 7000 DV inductively coupled plasma optical emission spectrometer (ICP-OES, Thermo Scientific, Waltham, MA, USA).

### 3.5. Electrochemical Measurement

T All electrochemical measurements were conducted on a CHI 660E electrochemical workstation (Shanghai Chenhua Instrument Co., Shanghai, China) using a three-electrode system in an H-type electrolytic cell. The as-prepared sample (1 cm × 1 cm) and an Ag/AgCl electrode served as the cathode and reference electrode in the cathode chamber, respectively, while a Pt sheet (1 cm × 1 cm) was used as the anode. The Ag/AgCl potential was converted to the reversible hydrogen electrode (RHE) scale using the Nernst equation:

*E_RHE_* = *E_Ag/AgCl_* + 0.059 × *pH* + 0.197

Linear sweep voltammetry (LSV) was performed over a potential range of −1.6 to 0 V versus Ag/AgCl. Electrochemical impedance spectroscopy (EIS) was conducted in 0.5 M Na_2_SO_4_ containing 0.01 M KNO_3_ over a frequency range of 0.01–100,000 Hz with a 5 mV AC amplitude and two points per decade. Cyclic voltammetry (CV) was carried out in the same electrolyte between −0.1 and 0.1 V versus Ag/AgCl at scan rates of 5–25 mV s^−1^, with 5 mV s^−1^ intervals, to estimate the double-layer capacitance (C_dl_) of the samples.

To estimate the eNO_3_^−^RR performance of the as-prepared samples, 0.01 M KNO_3_ in 0.5 M Na_2_SO_4_ was used as the electrolyte. Chronoamperometry (i–t) measurements were performed for 30–60 min at constant applied potentials to determine the optimal operating voltage for ammonia production. After electrolysis, the catholyte was collected for subsequent analysis. The ammonia yield was calculated using the following equation:

$${\mathrm{NH}}_{3}\mathrm{Yield}=\frac{C({NH}_{3})\times V}{S\times t}$$

$${{\mathrm{NO}}_{2}}^{-}\;\mathrm{Yield}=\frac{C({NO}_{2}^{-})\times V}{S\times t}$$

where C (NH_3_) is the measured concentration of NH_3_–N in the electrolyte, C(NO_2_^−^) is the measured concentration of NO_2_^−^-N, V (50 mL) is the volume of the catholyte, t (3600 s or 1800 s) is the electrolysis time, and S (1 cm × 1 cm) is the geometric surface area of the working electrode. The Faraday efficiency (FE) was calculated using the following equation:

$$\mathrm{FE}({\mathrm{NH}}_{3})=\frac{8\times F\times C({NH}_{3})\times V}{17\times Q}\times 100\%$$

$$\mathrm{FE}({{\mathrm{NO}}_{2}}^{-})=\frac{F\times C({NO}_{2}^{-})\times V}{46\times Q}\times 100\%$$

where F is Faraday’s constant (96,485 C mol^−1^), and Q is the total charge across the electrolyte.

The energy consumption for NH_3_ production was estimated according to

$${EC}_{{NH}_{3}}=\frac{8FU}{3.6\times 10^{6}M_{NH_{3}}FE_{NH_{3}}}$$

where F is the Faraday constant, U is the applied potential input, $M_{{NH}_{3}}$ is the molar mass of NH_3_, and ${FE}_{{NH}_{3}}$ is the Faradaic efficiency of NH_3_.

## 4. Conclusions

In summary, a Cu-MOF-derived, nano-dendritic, self-supported electrode on copper foam (CF@CNMD) was developed via a two-step strategy, which involved in situ electrosynthesis of Cu-MOF with CTAB as a structure-directing agent to control the three-dimensional architecture of the precursor, followed by electroreduction to form interconnected dendrite-like nanostructures. The CTAB-assisted CF@CNMD-C electrode exhibited a high electrochemically active surface area and enhanced interfacial charge-transfer capability. Electrochemical nitrate reduction tests confirmed its superior NO_3_RR activity. At −1.3 V vs. Ag/AgCl in a 1 h chronoamperometric test, the CF@CNMD-C electrode achieved a Faradaic efficiency of 89.6% and an NH_3_ yield rate of 5.004 mg h^−1^ cm^−2^. Increasing the potential to −1.7 V vs. Ag/AgCl in a 0.5 h test further enhanced its performance, delivering a Faradaic efficiency of 90.94% and an NH_3_ yield rate of 14.32 mg h^−1^ cm^−2^. The outstanding eNO_3_RR performance is attributed to the enlarged active surface area and accelerated charge-transfer kinetics. This work provides a novel strategy for constructing Cu-MOF-derived self-supported electrodes that combine structural stability with high catalytic activity, offering a sustainable approach for both ammonia synthesis and nitrate remediation.

## Data Availability

Data are contained within the article.

## Supplementary Materials

The following supporting information can be downloaded at https://www.mdpi.com/article/10.3390/molecules31132307/s1, Figure S1. SEM image of CF@Cu-MOF and CF@CNMD; Figure S2. SEM image of CF@Cu-MOF-C and CF@CNMD-C; Figure S3. EDS spectrum of the CF@CNMD-C electrode; Figure S4. EDS elemental mapping image of CF@CNMD-C; Figure S5. Survey scan XPS spectra of CF@CNMD and CF@CNMD-C; Figure S6. Deconvoluted C1s core-level XPS spectra of the CF@CNMD and CF@CNMD-C electrode; Figure S7. Deconvoluted O1s core-level spectra of CF@CNMD and CF@CNMD-C; Figure S8. CV curves of copper foam at different scan rates; Figure S9. Chronopotentiometry curve of CF@CNMD-C, CF@CNMD, and CF; Figure S10. Standard curves of NH_4_^+^ and NO_2_^−^ ion concentrations; Figure S11. The yield rate of NO_2_^−^ for electrodes during an hour of chronoamperometric electrolysis; Figure S12. Faraday efficiency of NO_2_^−^ for electrodes during an hour of chronoamperometric electrolysis; Figure S13. SEM images of CF@CNMD-C before and after 10 consecutive cycling tests; Figure S14. XRD patterns of CF@CNMD-C before and after 10 consecutive cycling tests; Table S1. Comparison of the eNO_3_-RR performance and energy consumption with other electrocatalysts at 0.01M; Table S2. Cu leaching concentration determined by ICP-OES analysis. Refs [61,62,63,64,65,66,67,68,69,70,71,72,73] are cited in Supplementary Materials.
