# Supplementary material for: Cu-MOF-Derived Nano-Dendritic Self-Supported Electrodes for Efficient Electrochemical Nitrate-to-Ammonia Conversion
Source: Molecules. 2026 Jul 1;31(13):2307. doi: 10.3390/molecules31132307 (PMC13363652; doi:10.3390/molecules31132307)
Supplement: Supplementary file 1 [file molecules-31-02307-s001.zip › molecules-4379105-supplementary.pdf]

Supplementary Information

Cu-MOF-Derived Nano-Dendritic Self-Supported Electrodes for Efficient Electrochemical Nitrate-to-Ammonia Conversion

Linfeng Qi, Yuan Gao, Xiangyan Zhong, Yunxiang Liang, Shijing Yuan, Yuan Wang*

Low-carbon Technology & Chemical Reaction Engineering Lab, College of Chemical Engineering, Sichuan University, Chengdu 610065, P. R. China
*Corresponding author: ysj@scu.edu.cn (S.Y.); Tel/Fax: +86-28-85405201

S1. Supplementary Experimental

S1.1 Determine of NO_2^--N ion concentration

First, Griess reagent was prepared, 0.2 g of naphthalene ethylenediamine hydrochloride, 2 g of sulfonamide, 5.88 ml of phosphoric acid were fixed to 100 ml with deionized water.8 groups of standard concentration of $NaNO_2$ solution were prepared with the concentration distribution of 0, 0.2, 0.5, 1, 1.5, 2, 3, and 4 ug ml^{-1}.1ml of Griess, 2ml of deionized water were added to the reagent respectively. The standard concentration curve of nitrite was plotted by measuring the absorbance of the sample at 540 nm using a UV spectrophotometer. After diluting the solution to be tested to reach the measurable concentration range, the sample to be tested was configured using the above method, the absorbance corresponding to the sample at 400 nm-700 nm was measured, and the absorbance corresponding to 540 nm was taken to obtain the corresponding concentration according to the standard curve graph.

S1.2 Determine of NH_3-N Detection ion concentration

First, the colorant was prepared by taking 12.5 g of salicylic acid, 12.5 g of sodium citrate trihydrate, 10 g of 1 M NaOH and fixing it to 250 ml with deionized water, Then the oxidizing agent was prepared by taking 2.95 ml of Sodium hypochlorite (The

effective chlorine content is 5.2%) and fixing it to 100 ml with deionized water, Finally, the catalyst was prepared by taking 0.25 g of sodium nitroso ferrocyanide and fixing it to 25 ml with deionized water.9 groups of standard concentration of NH_4Cl solution with concentration distribution of 0, 0.2, 0.5, 1, 1.5, 2, 3, 4, 5 ug ml^{-1}. 2 ml of standard solution, 2 ml of colorant, 1 ml of oxidizing agent, 0.2 ml of catalyst were added to prepare standard samples respectively. The standard concentration curve of NH_4^+ was plotted by measuring the absorbance of the sample at 660 nm using UV spectrophotometer. After diluting the solution to be tested to reach the measurable concentration range, the sample to be tested was configured using the above method, the absorbance corresponding to the sample at 500 nm-800 nm was measured, and the absorbance corresponding to 660 nm was taken to obtain the corresponding concentration according to the standard curve graph.

S2. Supplementary Results

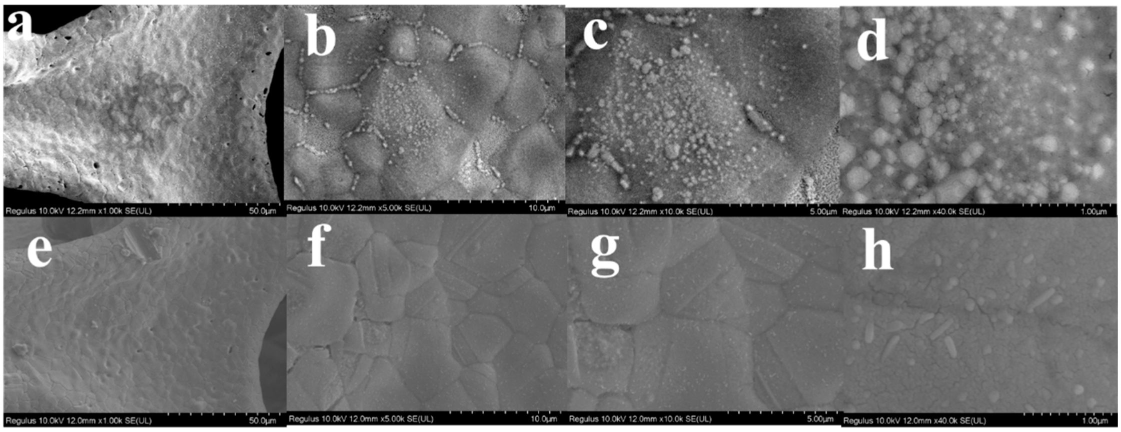

Figure S1 (a-d) SEM image of CF@Cu-MOF and (e-h) CF@CNMD electrodes

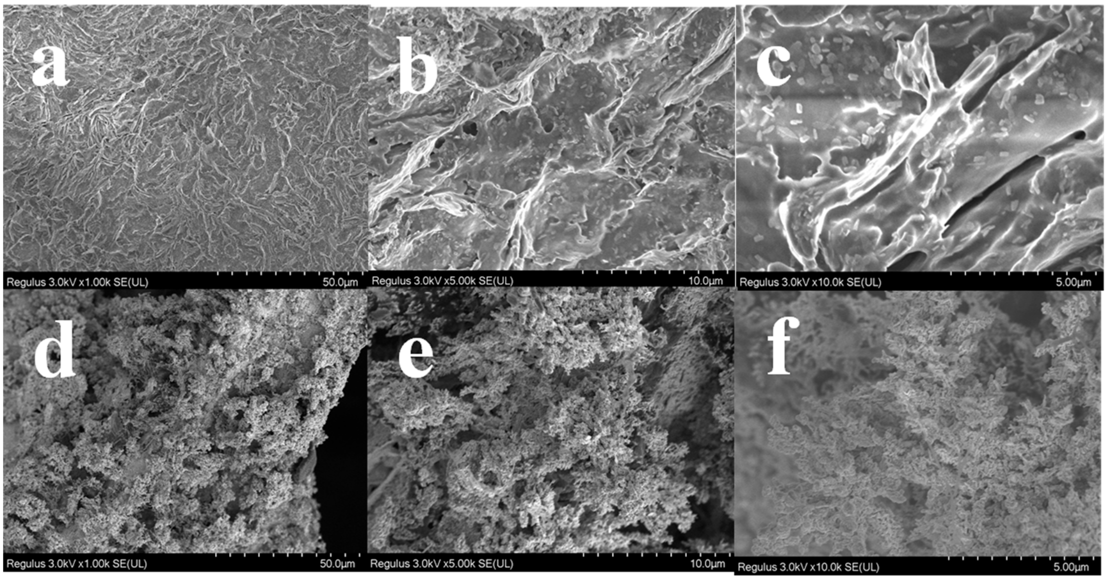

Figure S2. (a-c) SEM image of CF@Cu-MOF-C and (d-f) CF@CNMD-C

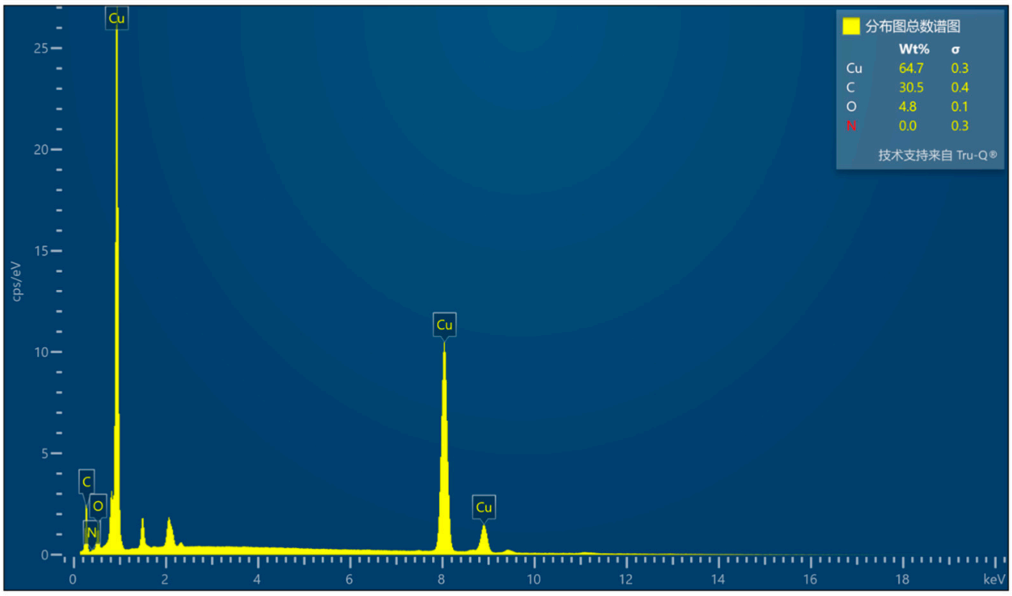

Figure S3. The EDS spectrum of the CF@CNMD-C electrode

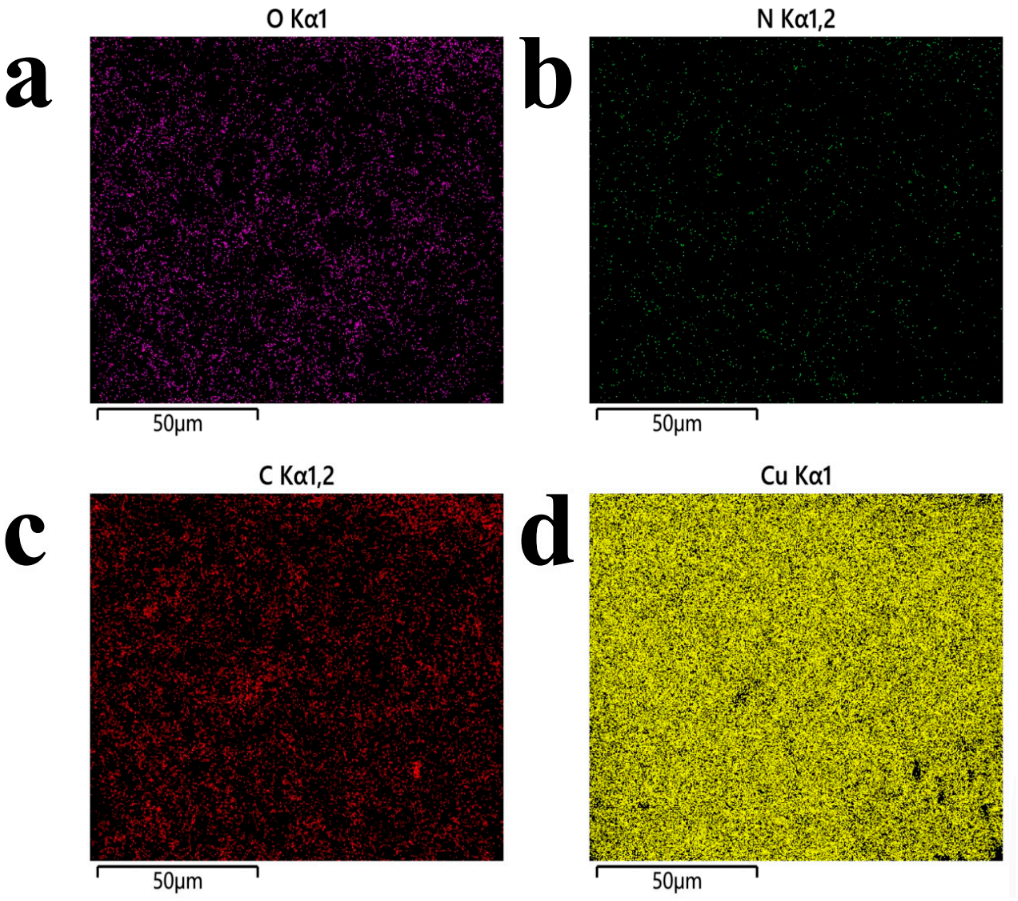

Figure S4. EDS elemental mapping image of (a) O, (b) N, (c) C, and (d) Cu

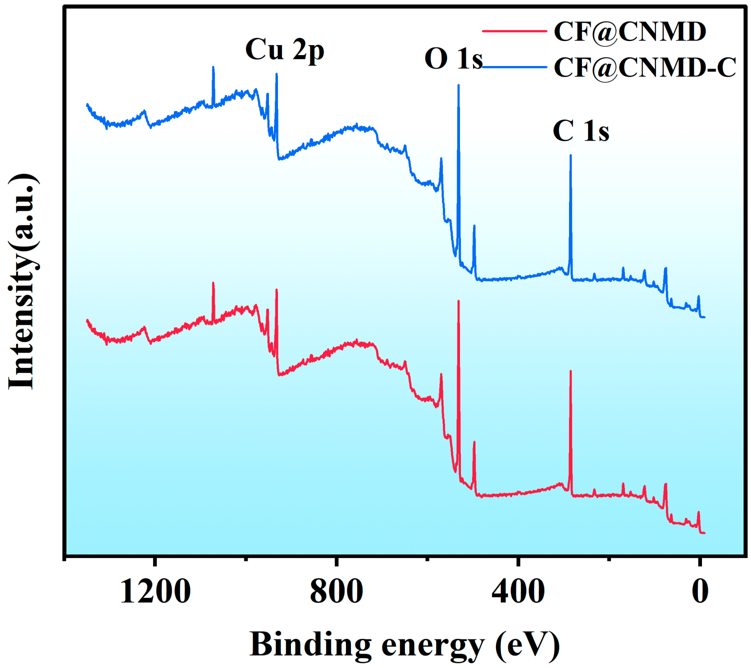

Figure S5. XPS survey scan spectra of the CF@CNMD and CF@CNMD-C electrodes

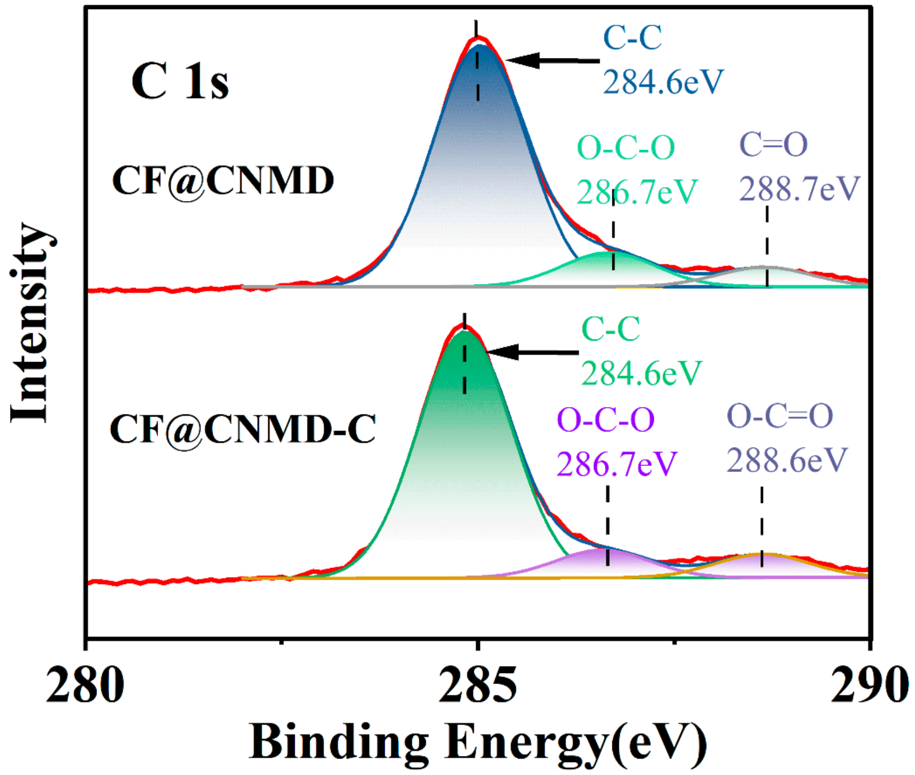

Figure S6. Deconvoluted C1s core-level XPS spectra of the CF@CNMD and CF@CNMD-C electrode

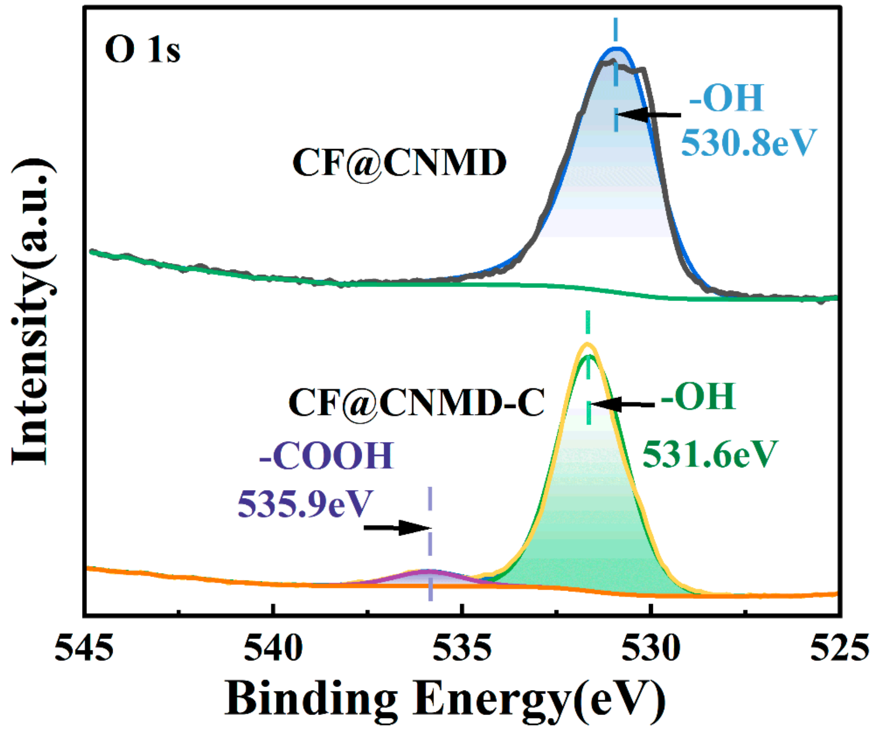

Figure S7. Deconvoluted O1s core-level XPS spectra of the CF@CNMD and CF@CNMD-C electrodes

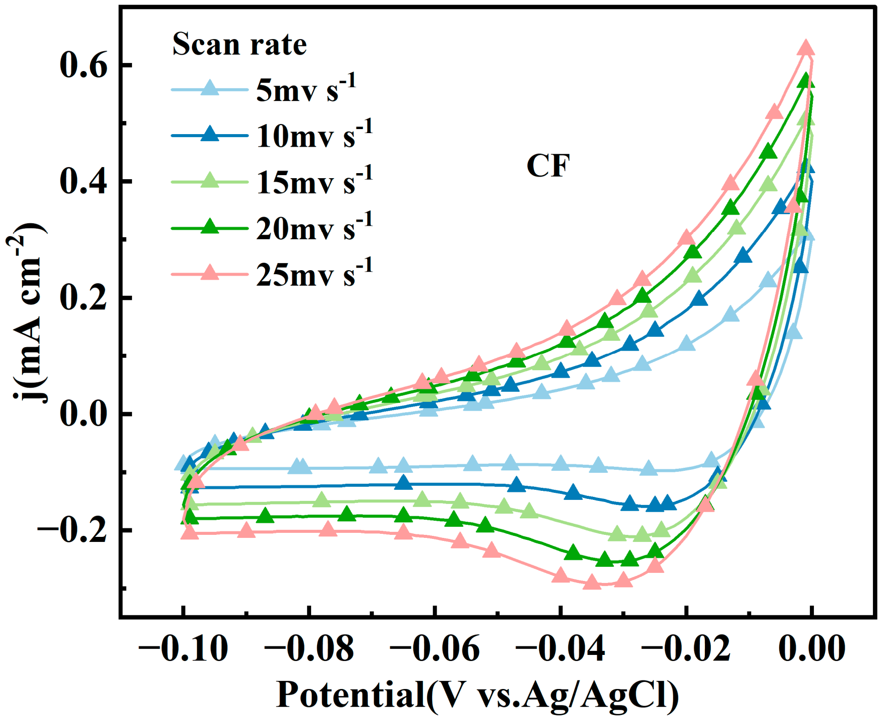

Figure S8. The CV curve of copper foam at different scan rates

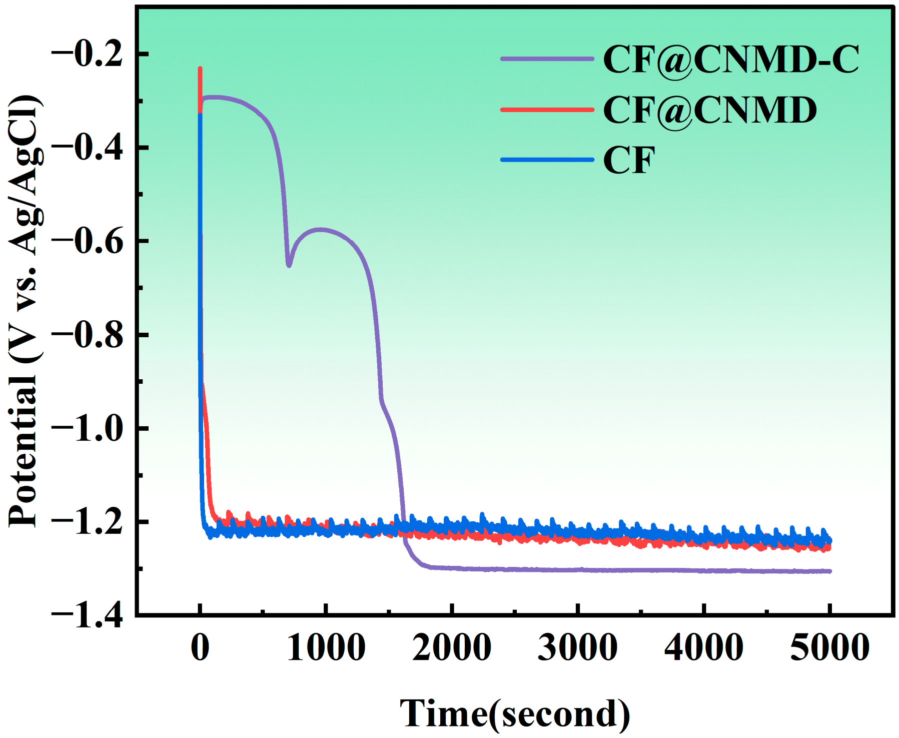

Figure S9. The chronopotentiometry curve of CF@CNMD-C, CF@CNMD and CF electrode in 0.5 M Na_2SO_4 containing 0.01 M KNO_3

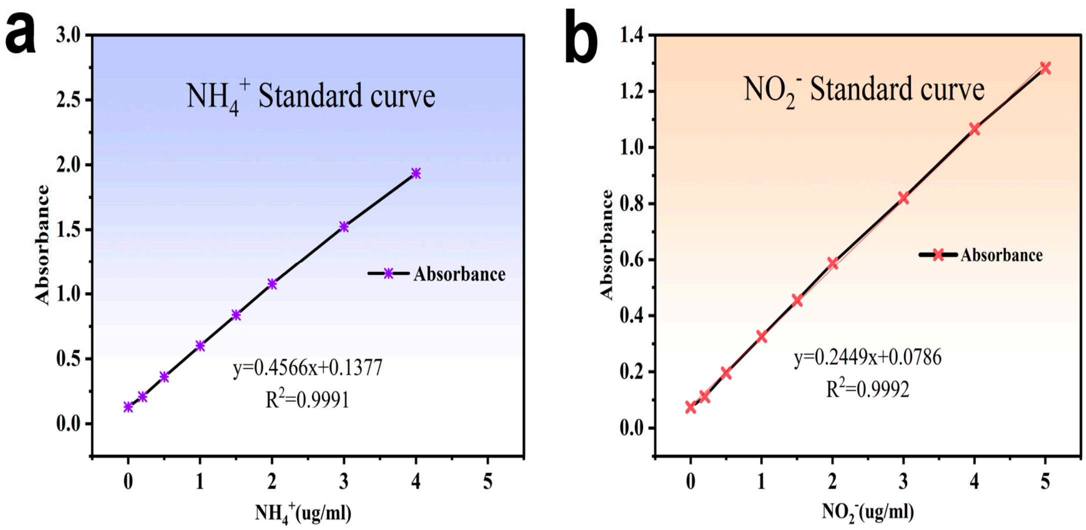

Figure S10 Standard curves of ion concentration for (a) NH_4^+ and (b) NO_2^-

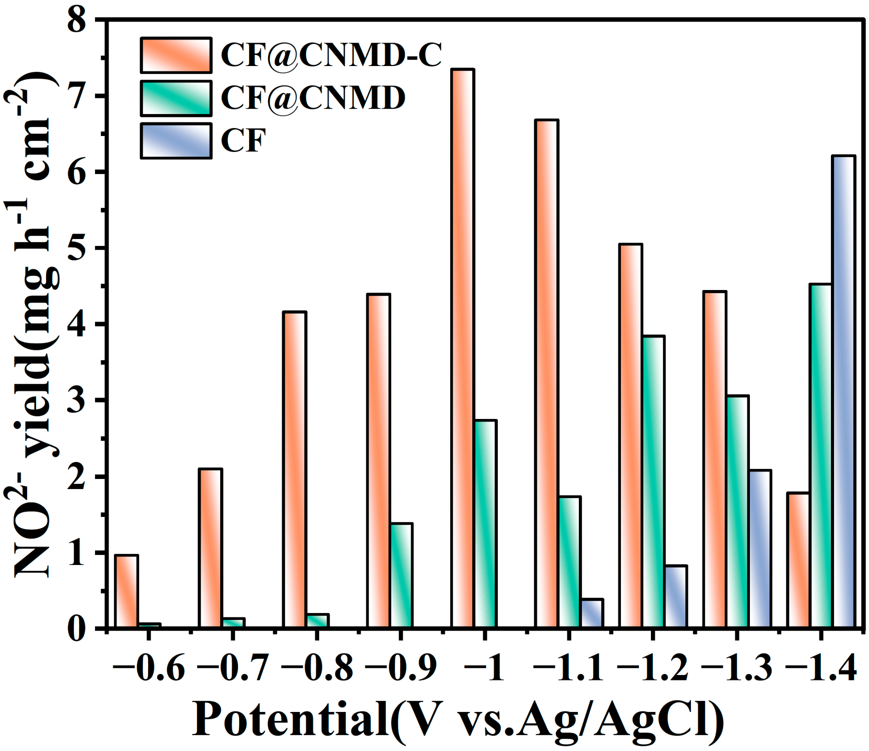

Figure S11. The NO_2^- yield of the CF@CNMD-C, CF@CNMD and CF electrodes during an hour chronoamperometric electrolysis.

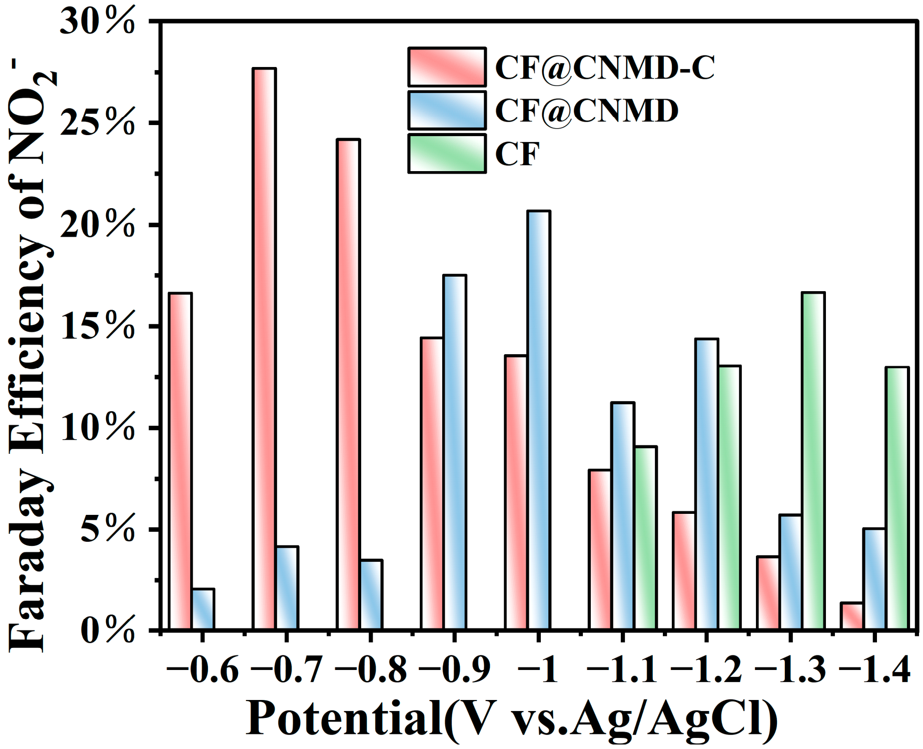

Figure S12. The Faraday efficiency of NO_2^- for the CF@CNMD-C, CF@CNMD and CF electrodes during an hour chronoamperometric electrolysis.

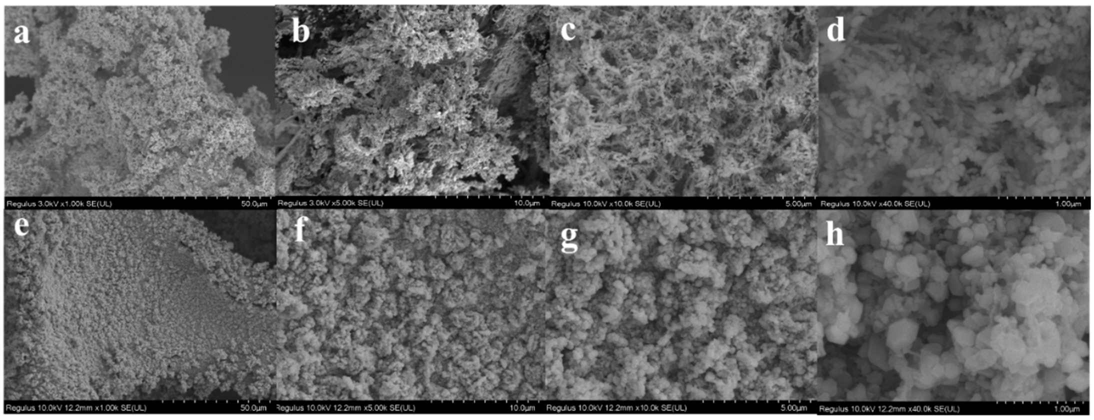

Figure S13. SEM images of CF@CNMD-C before and after 10 consecutive cycling tests.

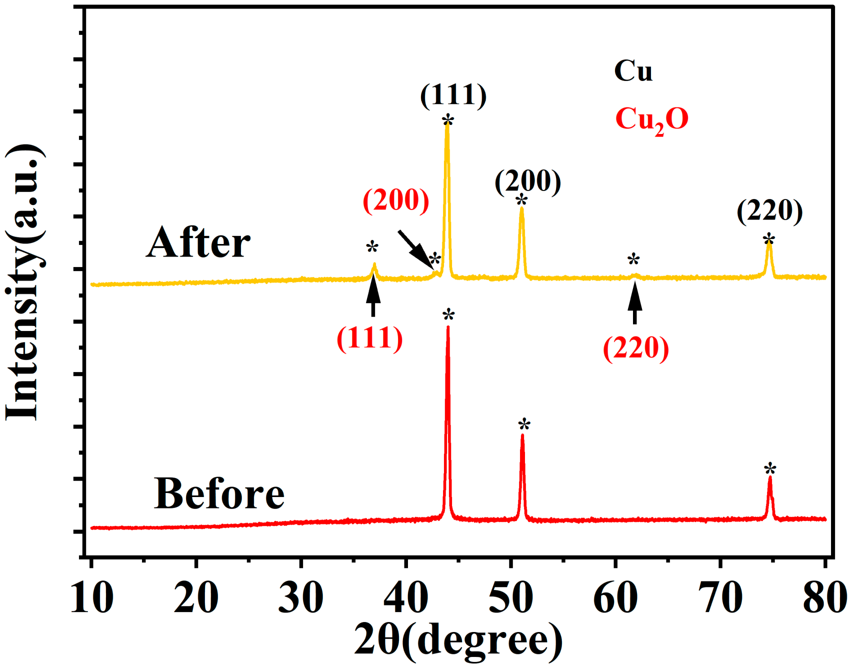

Figure S14. XRD pattern of CF@CNMD-C before and after 10 consecutive cycling tests.

Table S1. Comparison of the NO_3^-RR performance and energy consumption (EC) with other electrocatalysts at 0.01M

Electrocatalyst	Potential (V_{RHE})	FE (%)	NH_3 yield (mg/(h· cm))	EC (kWh/kg$_{NH_3}$)	Reference
**CF@CNMD-C**	**-1.09**	**88.13**	**13.94**	**15.58**	**This study**
CoO-Ru/CC	-1.00	93.41	2.502	13.48	[1]
Fe-CuO/Co_3O_4/C	−1.20	84.3	7.99	17.94	[2]
Cu-CoP	-1.00	85.10	7.65	14.79	[3]
Au-Cu NWs/CF	-1.05	84.10	5.336	15.72	[4]
Cu-nanodisks	−0.50	81.1	2.624	7.77	[5]
PdCu-nanospheres	−0.20	96.6	6.80	2.61	[6]
PdCu-nanocavities	−0.25	85.0	3.713	3.71	[7]
PdCuAg-nanotubes	−0.45	95.2	21.49	5.96	[8]
PdCu-nanospheres	−0.30	87.3	9.367	4.33	[9]
V_o-HCo_3O_4@SRGA	−0.80	96.5	1.858	10.45	[10]
$Cu_{2+1}O$/Ag-CC	-0.74	85.03	2.20	10.96	[11]
Co_3O_4-Fe_3O_4/C	−0.60	93.9	7.225	8.05	[12]
Cu/$Co_{0.85}$SeVSe	−0.60	93.5	2.36	8.09	[13]

Table S2. Cu leaching concentration determined by ICP-OES analysis.

Cycle times	Rep 1 (mg/L)	Rep 2 (mg/L)	Rep 3 (mg/L)	Mean (mg/L)	SD (mg/L)	RSD (%)	Cu amount in 50 mL/(μg)
1	0.229	0.211	0.209	0.216	0.0108	5.01	10.82
2	0.750	0.795	0.760	0.768	0.0236	3.07	38.41
3	0.962	0.866	0.844	0.891	0.0627	7.05	44.53
5	1.810	1.867	1.885	1.854	0.0392	2.11	92.70
8	0.935	0.813	0.704	0.817	0.1156	14.14	40.87
10	0.399	0.377	0.397	0.391	0.0122	3.11	19.55

Reference

[1] Zhao, J.; Liu, X.; Ren, X.; Zhao, D.; Li, Z.; Kang, Q.; Zhang, C.-W.; Hou, L.; Wei, Q.; Yuan, C. Nanoscale Ru unlocking nanoneedles assembled into hierarchical CoO microspheres for efficient nitrate-to-ammonia electroconversion. Chem. Sci. 2026, 17, 6165–6177.

[2] Zhang, B.; Zhao, J.; Qiu, H.; Chen, M.; Ren, X.; Wang, H.; Wei, Q. Boosting electrochemical nitrate reduction to ammonia by Fe-doped CuO/Co_3O_4 nanosheet/nanowire heterostructures. ChemPhysChem, 2024, 25, e202400738.

[3] Yang, W.; Chang, Z.; Yu, X.; Wu, P.; Shen, R.; Wang, L.; Cui, X.; Shi, J. Cu-Co dual sites tandem synergistic effect boosting neutral low concentration nitrate electroreduction to ammonia. Adv. Sci. 2025, 12, 2416386.

[4] Zha, Y.; Liu, M.; Wang, J.; Feng, J.; Li, D.; Zhao, D.; Zhang, S.; Shi, T. Electrochemical ammonia synthesis by reduction of nitrate on Au doped Cu nanowires. RSC Adv. 2023, 13, 9839–9844.

[5] Wu, K.; Sun, C.; Wang, Z.; Song, Q.; Bai, X.; Yu, X.; Li, Q.; Wang, Z.; Zhang, H.; Zhang, J.; Tong, X.; Liang, Y.; Khosla, A.; Zhao, Z. Surface reconstruction on uniform Cu nanodisks boosted electrochemical nitrate reduction to ammonia. ACS Mater. Lett. 2022, 4, 650–656.

[6] Sun, L.; Lv, H.; Xiao, J.; Liu, B. Enzymatic mesoporous metal nanocavities for concurrent electrocatalysis of nitrate to ammonia coupled with polyethylene terephthalate upcycling. Adv. Mater. 2024, 36, 2402767.

[7] Sun, L.; Yao, H.; Jia, F.; Wang, Y.; Liu, B. Intermediate confinement for selective ammonia electrosynthesis from nitrate on robust mesoporous metal catalysts. Adv. Energy Mater. 2023, 13, 2302274.

[8] Sun, L.; Yao, H.; Wang, Y.; Zheng, C.; Liu, B. Mesostructures engineering to promote selective nitrate-to-ammonia electroreduction. Adv. Energy Mater. 2023, 13, 2303054.

[9] Min, X.; Liu, B. Microenvironment engineering to promote selective ammonia electrosynthesis from nitrate over a PdCu hollow catalyst. Small 2023, 19, 2300794.

[10] Wang, K.; Zhao, T.; Zhang, S.; Wang, R.; Wang, M.; He, Z.; Ho, S.-H. Asymmetric defective site-triggered triple synergistic modulation in nanoconfined aerogel for superior electrochemical conversion of low-concentration nitrate into ammonia. Green Chem. 2025, 27, 6796–6803.

[11] Du, Z.; Yang, K.; Du, H.; Li, B.; Wang, K.; He, S.; Wang, T.; Ai, W. Facile and scalable synthesis of self-supported Zn-doped CuO nanosheet arrays for efficient nitrate reduction to ammonium. ACS Appl. Mater. Interfaces 2023, 15, 5172–5179.

[12] Liu, Y.; Zhong, X.; Liu, M.; Zhao, H.; Wang, Z.; Ni, R.; Wang, Y.; Yang, J.; Gao, F.; Li, Y.; Yuan, E.; Yuan, A.; Shi, W.; Yang, F. Composition-engineered FeCo nanoalloys with lattice

expansion and optimized electron structure boosting electrocatalytic nitrate reduction. Appl. Catal. B- Environ. Energy 2024, 355, 124205.
[13] Gu, Z.; Zhang, Y.; Wei, X.; Duan, Z.; Gong, Q.; Luo, K. Intermediates regulation via electron-deficient Cu sites for selective nitrate-to-ammonia electroreduction. Adv. Mater.2023, 35, 2303107.
